# Effect of mcl-PHA synthesis in flax on plant mechanical properties and cell wall composition

**DOI:** 10.1007/s11248-018-0105-y

**Published:** 2018-11-27

**Authors:** Magdalena Wróbel-Kwiatkowska, Mateusz Kropiwnicki, Jacek Żebrowski, Athanasios Beopoulos, Lucyna Dymińska, Jerzy Hanuza, Waldemar Rymowicz

**Affiliations:** 1 Department of Biotechnology and Food Microbiology, Faculty of Biotechnology and Food Sciences, Wrocław University of Environmental and Life Sciences, Chełmońskiego St. 37, 51-630 Wrocław, Poland; 2 Department of Plant Physiology, Faculty of Biotechnology, University of Rzeszów, Rzeszów, Poland; 3 Micalis Institute, INRA, AgroParisTech, Université Paris-Saclay, 78350 Jouy-en-Josas, France; 4 Department of Bioorganic Chemistry, Institute of Chemistry and Food Technology, Faculty of Engineering and Economics, Wrocław University of Economics, Komandorska Str. 118/120, Wrocław, Poland; 5 Institute of Low Temperatures and Structure Research, Polish Academy of Sciences, Okólna Str.2, Wrocław, Poland

**Keywords:** Flax, PHA (polyhydroxyalkanoates), Mechanical properties, Fatty acids, FTIR (Fourier transform infrared spectroscopy)

## Abstract

The high demand for new biomaterials makes synthesis of polyhydroxyalkanoates (PHA) in plants an interesting and desirable achievement. Production of polymers in plants is an example of application of biotechnology for improving the properties of plants, e.g. industrial properties, but it can also provide knowledge about plant physiology and metabolism. The subject of the present study was an industrially important plant: flax, *Linum usitatissimum* L., of a fibre cultivar (cv Nike). In the study the gene encoding PHA synthase from *Pseudomonas aeruginosa*, fused to a peroxisomal targeting signal, was expressed in flax plants with the aim of modifying the mechanical properties of plants. Medium-chain-length (mcl) hydroxy acids in flax plants from tissue cultures were detected by GC-FID and FTIR method. The introduced changes did not affect fatty acid content and composition in generated flax plants. Since mcl-PHA are known as elastomers, the mechanical properties of created plants were examined. Modified plants showed increases in the values of all measured parameters (except strain at break evaluated for one modified line). The largest increase was noted for tensile stiffness, which was 2- to 3-fold higher than in wild-type plants. The values estimated for another parameter, Young’s modulus, was almost at the same level in generated flax plants, and they were about 2.7-fold higher when compared to unmodified plants. The created plants also exhibited up to about 2.4-fold higher tensile strength. The observed changes were accompanied by alterations in the expression of selected genes, related to cell wall metabolism in line with the highest expression of *phaC1* gene. Biochemical data were confirmed by spectroscopic methods, which also revealed that crystallinity index values of cellulose in modified flax plants were increased in comparison to wild-type flax plants and correlated with biomechanical properties of plants.

**Electronic supplementary material:**

The online version of this article (10.1007/s11248-018-0105-y) contains supplementary material, which is available to authorized users.

## Introduction

Polyhydroxyalkanoates (PHA) are natural origin, thermoplastic polyesters made up of (*R*)-3-hydroxy fatty acid monomers and synthesized by various bacteria species for energy and carbon storage, under nutrient limitation conditions (Ryu et al. 1997; Valappil et al. 2008). Polyhydroxyalkanoates, in general, are considered as an alternative to synthetic, petroleum based plastics, due to their similar properties, biodegradability, and ability to be made from renewable sources (Braunegg et al. 2004).

Nowadays many researchers are focused on the synthesis of polyhydroxyalkanoates (PHA) in bacteria (Nomura et al. 2004; Davis et al. 2013), yeast (Zhang et al. 2006), algae (Chaogang et al. 2010) and plants. Various plants are under investigation, including model plants such as *Arabidopsis thaliana* (Poirier et al. 1992; Kourtz et al. 2007) and crop plants, e.g. potato (Bohmert et al. 2002; Romano et al. 2005), tobacco (Nakashita et al. 2001; Arai et al. 2001; Suzuki et al. 2002) and rape (Houmiel et al. 1999; Valentin et al. 1999).

Attempts to produce these polymers in trees, i.e. poplar *Populus* ssp. (Dalton et al. 2011) and *Tamarix* (Endo et al. 2006), have also been successful.

Industrially important crop plants—cotton and flax—have been used for PHA synthesis, too (John and Keller 1996; Wróbel-Kwiatkowska et al. 2007).

Different systems of transgene expression for PHA genes have been applied, aiming to achieve efficient polymer production, i.e. constitutive promoters (such as the 35S CaMV promoter), inducible promoters, e.g. ecdysone inducible promoter (Kourtz et al. 2007), promoters expressed in specific tissue, for example in phloem tissue (Wróbel et al. 2004), etc. Diverse cell compartments have also been used for polymer synthesis, e.g. cytosol (Poirier et al. 1992; Endo et al. 2006), chloroplasts (Valentin et al. 1999; Bohmert et al. 2000; Wróbel et al. 2004) and peroxisomes (Poirier et al. 1999; Tilbrook et al. 2011).

In the present study the *phaC1* gene, coding PHA synthase from *Pseudomonas aeruginosa*, was constitutively over-expressed in flax plants. Flax is an annual crop plant, cultivated in temperate climates for either fibre or oil, depending on the cultivar. In the case of fibrous plants, the main product is fibre derived from phloem tissue. A recent development in transcriptome analysis showed that even mature flax fibres, which deposited a tertiary cell wall, were still transcriptomic and metabolically active (Gorshkov et al. 2017). Thus flax fibres are under investigations in basic research (Ibragimova et al. 2017; Gorshkov et al. 2017) as well as in industrial applications (Holbery and Houston 2006). Currently there is increasing demand for natural fibres, which are more healthy than synthetic fibres, and they exhibit biocompatibility when they are used as a component of composite materials (Wróbel-Kwiatkowska et al. 2017).

In the present research a construct containing the *phaC1* gene (from *P. aeruginosa*), fused to the isocitrate lyase peroxisomal targeting sequence from *Brassica napus* and expressed under the 35S CaMV promoter, was used for transformations of flax plants (fibrous cultivar). Plant peroxisomes are dynamic organelles, especially as concerns metabolism (Hu et al. 2012). Previous studies showed that intermediates from beta-oxidation of fatty acids in peroxisomes may be substrates for PHA production (Langenbach et al. 1997; Poirier 2002). It was also demonstrated that polymer synthesis in peroxisomes does not affect plant phenotype (Mittendorf et al. 1998).

In contrast to the enzyme derived from *Ralstonia eutropha* (now called *Cupriavidus necator*), which synthesizes scl (short chain length)-PHA, the enzyme from *P. aeruginosa* is able to produce mcl (medium chain length)-PHA, a polymer containing hydroxy acids from C6 to C14 (Reddy et al. 2003). The composition of the polymer influences its chemical and physical properties; thus scl-PHA (consist of C3 to C5 monomers) are known as more brittle polymers than mcl- polyhydroxyalkanoic acids, which are recognized as elastomers and possess highly elastic properties (Jiang et al. 2006; Cerrone et al. 2014). These polymers belong to the group of polyhydroxyalkanoates, which are also biodegradable (Schirmer et al. 1993) and biocompatible in animal tissue (Ali and Jamil 2016; Gredes et al. 2012) and they can serve as a source of new biomaterials for medicine (Rai et al. 2011). Thus the main aim of the present study was to generate flax plants with over-expression of the PHAC1 synthase gene from *P. aeruginosa* and to characterize created plants in terms of their biochemical, mechanical and spectroscopic properties. We used a fibrous cultivar of flax to evaluate in the future the influence of introduced modifications on potential industrial application of fibres isolated from these plants.

## Materials and methods

### Plant material and growth conditions

A fibrous cultivar of flax (*Linum usitatissimum* L. cv. Nike) was chosen for the experiments and grown in tissue culture in a phytotron under a 16 h light (21 °C)/8 h dark (16 °C) regime. The seeds of flax were germinated and then grown on MS medium (Murashige and Skoog 1962) supplemented with 1% sucrose and 0.8% agar, medium was adjusted to pH 5.8 before autoclaving (121 °C, 20 min). PPM™ Plant Preservative Mixture (Plant Cell Technology) at a concentration of 750 μL/1 L was added to the medium. The seeds were treated with 50% (v/v) solution of PPM™ in the aim of disinfection (Thomas et al. 2017), after rinsing with sterile water, the seeds were germinated in the darkness during 7–14 days. Young flax seedlings were used for genetic transformation via *Agrobacterium*-mediated technique.

### Transformation and screening of flax plants

**Fig. 1:**
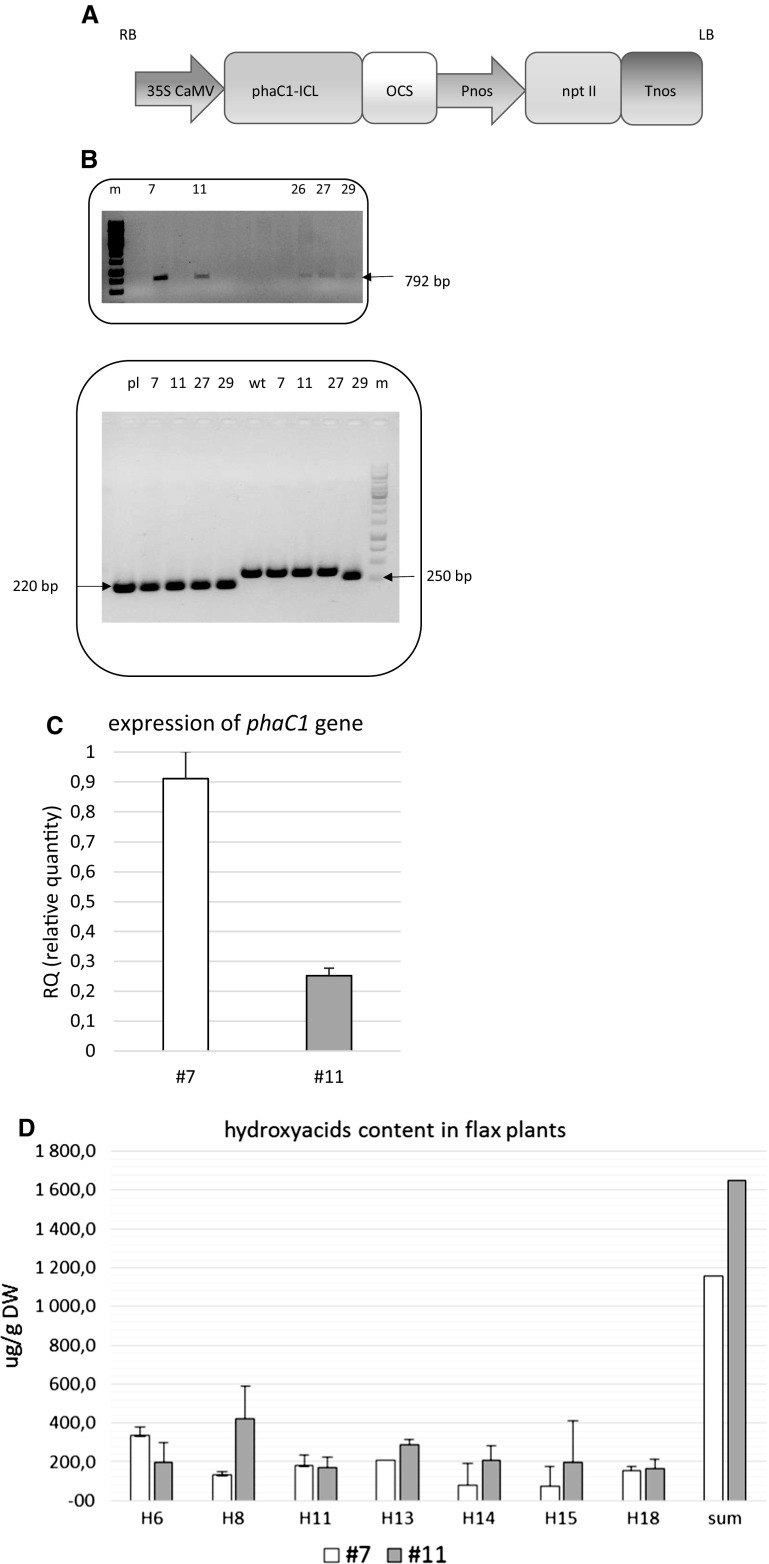
**a** Schematic illustration of *phaC1* cassette used for flax transformation. T-DNA contained: 35S CaMV- cauliflower mosaic virus promoter; phaC1, *phaC1* synthase gene from *P. aeruginosa*; ICL-34 amino acids sequence of the *Brassica napus* isocitrate lyase targeting to peroxisomes; ocs, octopine synthase gene terminator; Pnos, nopaline synthase promoter; npt II, neomycin phosphotransferase gene; Tnos, nopaline synthase gene terminator. RB, LB: right and left border sequence of the T-DNA. **b** Selection of generated plants by PCR method. Top panel: Fragment of 792 bp of *phaC1* gene from *P. aeruginosa* was amplified in PCR reaction on genomic DNA, isolated from plants. Integration of introduced gene was confirmed for 5 transgenic lines. Bottom panel: Fragment of 35S CaMV promoter (220 bp; first 5 lanes) and highly conserved region of chloroplast DNA (297 bp; last 5 lanes) were amplified with the use of specific primers. Genomic DNA was isolated from plants, cultivated in tissue cultures. pl, plasmid used for transformation (pART27-phaC1); wt, genomic DNA isolated from control, wild-type plants; different transgenic lines are numbered; m, marker 1 kb ladder. **c** Expression of *phaC1* gene in selected plants (numbered 7 and 11). The total RNA was isolated from tissue-cultured flax plants and used for cDNA synthesis. Then semi-quantitative PCR reaction was carried out on the cDNA template. Quantitative data were normalized with expression of the actin gene, n = 3. **d** Determination of mcl-PHA isolated from tissue cultured flax plants, obtained after transformation with *phaC1* gene. The level of PHA monomers was determined as described in the “Materials and methods” section by gas chromatography–flame ionization detection (GC-FID) method. The results derived from 3 samples analyzed per each line

The procedure of genetic flax transformation and regeneration applied in this study was described previously by Wróbel-Kwiatkowska et al. (2007) and Lorenc-Kukuła et al. (2007). After inoculation of *Agrobacterium tumefaciens* the flax explants were subsequently incubated on callus and shoot induction medium, after that the regenerated plants were rooted in classical MS medium with 1% sucrose, described in “Plant material and growth conditions” section. The plasmid for genetic transformation was kindly provided by Prof. Poirier (Mittendorf et al. 1998). The construct contained gene for PHA synthase (*phaC1*) from *P. aeruginosa* under the 35S CaMV promoter (Fig. 1A), the gene was fused to the peroxisomal targeting signal from *B. napus* (isocitrate lyase peroxisomal targeting sequence). A binary vector (pART27) was introduced by electroporation into the *Agrobacterium tumefaciens* strain LBA 4404, which was then co-cultured with flax explants (cotyledons, hypocotyls). Flax plants regenerated on callus and shoot induction medium were pre-screened by the PCR method with primers specific for the used *phaC1* gene (F: 5′GCTGAACCTGAATCCGGTGA3′; R: 5′ACTACCTCGATGGCCTCCTT3′) and fragment of 35S CaMV promoter (F: 5′ GAAAAGGAAGGTGGCTCCTA3′, R: 5′GGTCTTGCGAAGGATAGTGG3′), as the template genomic DNA isolated from tissue-cultured plants was applied. Isolation of DNA was performed by Thermo Scientific Phire Plant Direct PCR Master Mix kit. The conditions of PCR method were as recommended in the protocol of the kit. The products of amplifications were electrophoretic separated in 0.8% agarose in the presence of ethidium bromide. The integration of the introduced gene in the genome of generated plants was investigated by detection of fragment of PHAC1 synthase gene (amplicon 792 bp) and 35S CaMV promoter (amplicon 220 bp). Additionally control primers, which amplify fragment of conserve region of chloroplast DNA (amplicon 297 bp) and supplied in PCR set, were applied in PCR reaction.

Preselected plants were used for further selection at the transcriptional level. Thus total RNA was isolated from created plants using the TRI Reagent method (as described in “Analysis of expression of selected genes in generated flax plants” section), and after removing DNA by digestion with DNase I, RNA was used for cDNA synthesis (dART RT kit, EURx). Synthesized cDNA was used as a template for semi-quantitative PCR analysis. The products of this reaction were determined by gel electrophoresis (0.8% agarose) and quantified by densitometry (BIO-GENE 11.9 software, Vilber).

### Analysis of expression of selected genes in generated flax plants

The expression of selected, investigated genes was determined by semi-quantitative RT-PCR. The total RNA was extracted from flax plants, derived from tissue cultures by TRI Reagent (Sigma-Aldrich) according to the manufacturer’s protocol, DNA was removed by DNase I (Thermo Scientific), then purified RNA was used as a template in the reverse transcription reaction using dART RT kit (EURx). Synthesized cDNA was used for PCR reactions conducted with DreamTaq Green DNA Polymerase (Thermo Scientific). The sequences of used primers were specified earlier for flax plants by Wojtasik et al. 2014. The house-keeping gene encoding actin served as a reference gene. PCR products were detected on agarose gel (0.8%) and quantified by densitometry analysis (BIO-GENE 11.9 software, Vilber). Obtained data were normalized with the expression of the actin gene in each sample.

### PHA and FA measurements in created flax plants

For the extraction of PHA, plant tissue from in vitro cultures was lyophilized, weighed and transferred to a glass tube. The dried material was extracted twice with warm methanol (65 °C, 1 h) to remove lipids, while PHA, which is insoluble in methanol, remained associated with the tissue. After centrifugation and removal of the residual methanol, the material was suspended in 1 mL of chloroform, to which 1 mL of methanol containing 15% sulfuric acid and 50 μg/mL of commercial C17:0 (Sigma) as an internal standard were added. The mixture was heated at 100 °C for 2.5 h and immediately cooled down on ice. 1 mL of 0.1% NaCl was added to the tube, and the mixture was vortexed vigorously and centrifuged at 3000*g* for 5 min. The chloroform phase, containing the methyl-esters of 3-hydroxy acids, was analyzed by a Varian 3900 gas chromatograph equipped with a flame ionization detector and a Varian Factor Four vf-23 ms column with a bleed specification at 260 °C of 3 pA (30 m, 0.25 mm, 0.25 μm). For the extraction of total lipids, exactly the same procedure was followed, omitting the methanol extraction phase.

### Determination of cellulose and lignin content in flax plants

The cellulose amount was measured by the anthrone method (Updegraff 1969) in tested flax plants from tissue cultures, while the lignin level was estimated via the acetyl bromide technique as described by Iiyama and Wallis (1990). The procedures of those methods were previously described in detail by Wróbel-Kwiatkowska et al. (2009).

### Stem tensile properties determined for generated flax plants

The 15 mm long stem sections of freshly harvested, fully hydrated plantlets were examined for mechanical properties in a tensile unidirectional test in the computer–driven Instron universal machine (model 5542, High Wycombe, UK). The specimens were cut from the basal part of the stems and glued at the ends to plastic sticks with a small amount of cyanoacrylate adhesive, which were firmly connected by means of grips to the load cell (10 N capacity) and to the immovable part of the instrument. All mechanical parameters were estimated on the basis of the load vs displacement relationship for the 1 mm/min rate of deformation. Sample strains were measured locally at high precision (1/100 pixel) on the basis of video recordings analyzed in post-recording mode using the Video Gauge system (Imetrum, UK). The displacement of selected points on the sample surface could be tracked at the accuracy of about 10 µm for a gauge length of about 0.5 mm. The use of a non-contact video extensometer allowed us to avoid the interfering effects of use of glue at the sample ends, sample slippage within clamps, and non-linearity in strain distribution close to the grids.

The stress–strain curve was the basis for mechanical parameter calculation. The relative strain [%] was calculated directly from the displacement of tracked points during tensile sample deformation. The strain at break was defined as (x_max_ − x_0_)·100%/x_0_, where (x_0_) was the distance between selected points before loading (the gauge length) and (x_max_) was the distance between the same points at the break. The tensile stiffness EA of the stem structure was determined as the ratio of the load increment to the corresponding relative strain at the linear phase of sample deformation. Young’s modulus E was calculated referring the EA to the unit cross-sectional area (A) of the sample (E = EA/A), where A was determined for fresh microscopic slices using the ImageJ (v.1.51f) software. The tensile strength was defined as the ratio of the load at break to the cross-sectional area (A).

### Spectroscopic studies

IR spectra of plant samples were measured at room temperature in the spectral range 400–4000 cm^−1^ using a Thermo Scientific Nicolet 6700 FT-IR spectrometer with 2 cm^−1^ resolution. The ATR-diamond equipment was used for recording the reflection spectra.

Raman spectra were measured with a Bruker RFS 100/S FT-Raman spectrometer (Bruker, Karlsruhe, Germany). A diode-pumped Nd:YAG laser at 1064 nm with an output of 400 mW was used as the excitation source. The spectral resolution was 2 cm^−1^ and 250 scans were collected.

Three replicates of IR and Raman spectra were recorded for every sample. The procedures for spectroscopic measurements were standardized. The same spectral parameters, the power of excitation source, the acquisition and accumulation were applied to the all studied samples.

The mathematical processing of the measured spectra was performed using the computer program ORIGIN 7.5. The crystallinity index Icr was calculated as the integral intensity ratio of the 1380 and 2920 cm^−1^ IR bands (Kruer-Zerhusen et al. 2018; Wróbel-Kwiatkowska et al. 2015).

## Results and discussion

### Transgenic plant generation and PHA determination in plants

Flax plants obtained after Agro-mediated transformation procedure (described in the Materials and Methods section) were pre-screened by the PCR method (Fig. 1b). A total of 158 flax explants were taken for genetic transformation, all of them developed callus tissue and the shoot regeneration frequency achieved 18.9%. Finally 30 transformants were generated and 5 of them exhibited transgene integration in genomic DNA, selection was performed via two PCR reactions. Thus fragment of introduced gene of phaC1 synthase was detected in plant genome (amplicon 792 bp), and fragment of 35S CaMV was investigated (Fig. 1b). In control reaction the primers, which amplify fragment of chloroplast DNA were applied (amplicon 297 bp).

Preselected plants were amplified in tissue cultures, for two of them numbered 7 and 11 the expression of the introduced gene at the transcriptional level was confirmed (Fig. 1c). Thus these two plants gave the two transgenic lines, which were the subject of present research. No differences in phenotype of plants from selected lines (#7 and 11) were observed when compared to non-transformed flax from tissue cultures. The introduced gene resulted in an increased level of PHA up to 1.6 mg/g DW (in line #11). This amount was higher than the PHA content in plants from line 7, which showed quite a different PHA profile (Fig. 1d). The dominant hydroxy acid in plants from line 11 was 3-hydroxyoctanoic acid (H8), while in plants from line 7 it was 3-hydroxyhexanoic acid (H6). It should also be pointed out that the amount of PHA did not exactly correspond to the level of gene expression for the introduced gene (*phaC1*), possibly due to the influence of the transgene location in the plant genome or other, unknown reasons.

### Quantitative analysis of fatty acids in generated flax plants

**Fig. 2:**
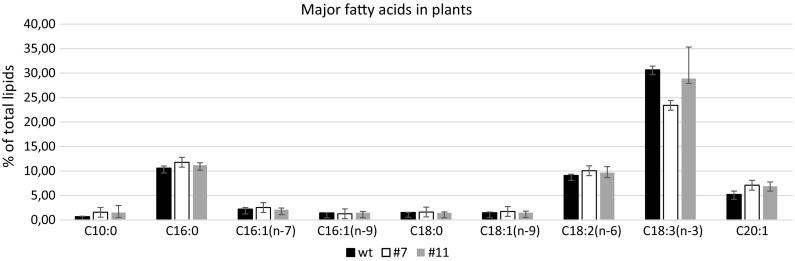
Fatty acids composition in green tissue of transgenic flax plants. The methyl esters of fatty acids were measured as described in the Materials and methods section. The statistical analysis was performed by Student’s t-test (*P < 0.05; **P < 0.01), n = 3

Since peroxisomes are the place of fatty acid peroxidation (Poirier et al. 2006; Goepfert and Poirier 2007) and PHA in selected lines may be synthesized in this compartment starting from β-oxidation intermediates, it was of interest to investigate whether the fatty acid profile in tested plants was influenced. Thus fatty acid methyl esters were determined in lyophilized green tissue of plants derived from in vitro cultures. The analyses confirmed that the major fatty acids in green tissue possessed 18 and 16 carbons; the most abundant fatty acid was linolenic acid C18:3, followed by linoleic acid C18:2 and palmitic acid C16:0 (Fig. 2). A reduction of the amount of linolenic acid was observed in both transgenic lines in comparison to wild-type plants, the highest decrease being observed for line 7, for which the highest expression of the introduced gene was observed. Observed changes were however not statistically significant. The level of linoleic and palmitic acid was slightly increased in both modified lines when compared to control plants. This tendency may reflect the hydrolysis of lipids in plant tissue or the rearrangements of fatty acids (Chu and Tso 1968). The amounts of stearic (C18:0), oleic (C18:1) and palmitoleic acids (C16:1, n-7) were almost at the same level as in the samples from control, unmodified flax plants. Two other fatty acids, capric acid (C10:0) and eicosenoic acid (C20:1), were at slightly increased levels in modified plants compared to wild-type plants.

### Mechanical properties of created flax plants

**Fig. 3:**
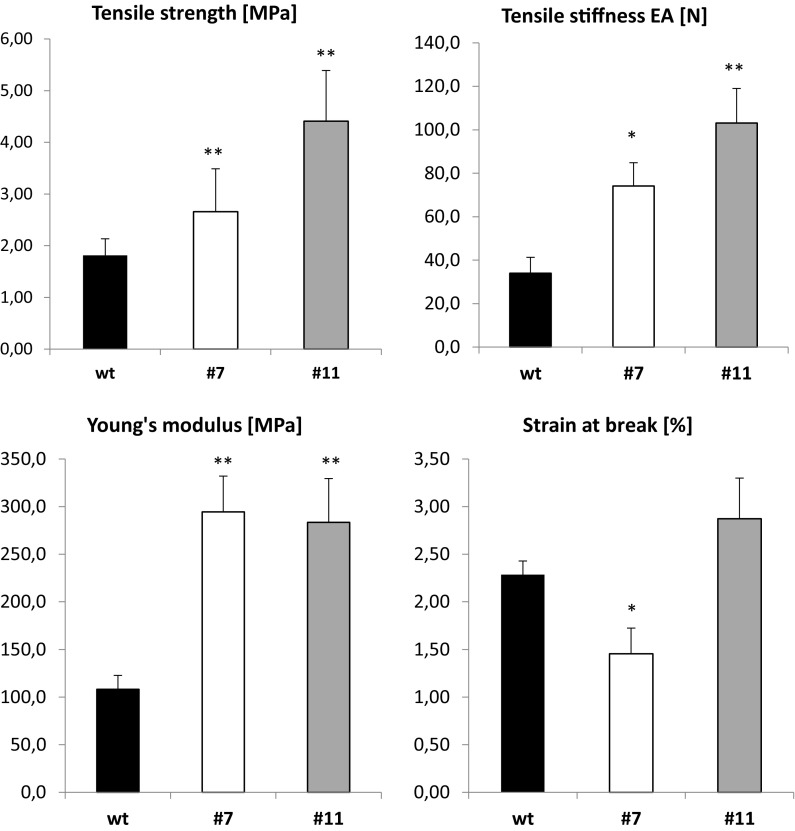
Mechanical properties of flax stems determined in tensile tests for wild-type flax plants cv. Nike and two genetically engineered lines (#7 and 11). The parameters were estimated as described in the “Materials and methods” section. The statistical analysis was performed by Student’s t-test (*P < 0.05; **P < 0.01), n = 3

PHA are known as elastomers; therefore generated flax plants were analyzed in terms of their mechanical properties (Fig. 3). The expression of the *phaC1* gene in flax plants caused an increase in the values of all measured mechanical parameters (except strain at break for one line, i.e. line 7) in comparison to non-transformed, wild-type plants. The tensile stiffness EA of the stem in the middle part, a quantity dependent on the cross-section area and tissue stiffness, was 2- to 3-fold higher in modified flax lines in comparison to control flax plants, and line 11 appeared superior relative to line 7. The difference between plants from both generated lines was reduced when they were compared for the values of the effective Young’s modulus, which was from 2.6-fold to 2.7-fold higher in plants from lines 11 and 7, respectively, than in control plants. This parameter, in the case of the highly heterogeneous material that is plant stem, may be affected by several factors including the contribution of fibre total area to stem cross-section, and the fibre mechanical quality. The latter might be determined by the rate of tissue differentiation and thus the accumulation of PHA and/or the cell wall composition. The effect of turgor was reduced, ensuring its maximum value by maintaining the samples high humidity until the measurements.

The tensile strength indicating the capacity of tissues to carry loads before failure also differentiated the examined plants. The highest value for this parameter was observed for line 11, and it was about 2.4-fold higher than that measured for control plants, while line 7 showed a medium value and it was about 40% higher than that estimated for wild-type plants. Interestingly, for plants from line 7 reduced strain at break was also observed when compared to wild-type plants. This may suggest occurrence of some imperfections in the structure of the fibre, the main loading compound, as a result of the introduced genetic modification. All considered, line 11 showed considerably improved mechanical properties with respect to all tested parameters. This line also revealed the highest mcl-PHA level, and thus measured mechanical properties of stems were proportional to the amounts of the sum of elastomers. These data were referred to the whole stem properties, and thus to a composite structure, where fibres contribute only partially. Establishing the effect of the genetic manipulation on the mechanical performance of mature fibres requires, however, further studies in future.

### Analysis of cell wall compounds in modified plants

The observed improved mechanical properties of modified plants led us to check the level of cell wall components in generated flax plants. The cell wall determines tissues’ mechanical properties (Whitney et al. 1999), particularly in cells which have completed their elongation and undergo differentiation through deposition of cellulose and lignin. Cellulose is the main load-bearing compound (Li et al. 2003), affecting the wall tensile properties proportionally to its content and in dependence of its spatial arrangement (microfibril angle). In turn, lignin accumulating in differentiating tissues contributes primarily to compressive mechanical properties of the cell walls and tissues. However, it may also affect tensile properties of young tissues filling empty wall spaces and contributing to cross-linking cellulose microfibrils. Both polymers were quantitatively evaluated for all examined plant types.

**Fig. 4:**
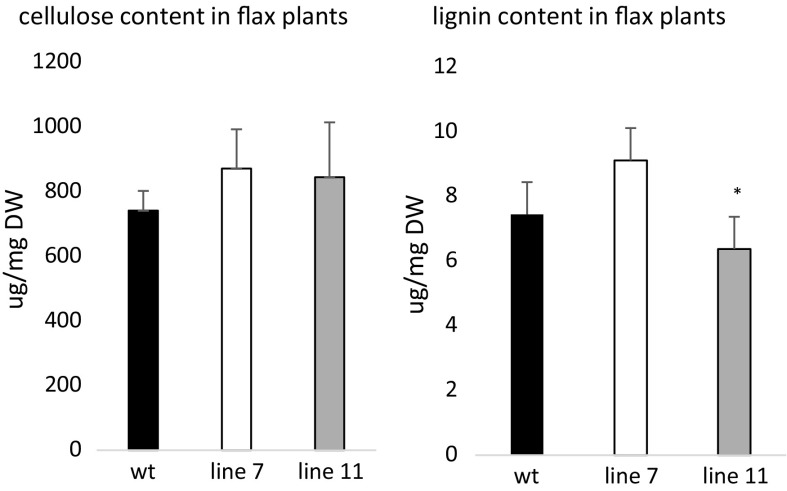
Cellulose and lignin level in tested flax plants, obtained after transformation with *phaC1* gene. Analysis was performed as described in the “Materials and methods” section. The level of cellulose and lignin was determined in plants derived from tissue cultures. The statistical analysis was performed by Student’s t-test (*P < 0.05; **P < 0.01), n = 3

In both transgenic lines an elevated amount of cellulose was found when compared to wild-type plants, while the cellulose quantity was almost at the same level in both modified lines (it should be pointed out that these changes were, however, not statistically important). The only statistically significant alteration was a reduction of lignin amount in line 11 (Fig. 4). The lignin content corresponded well with the strain values at break, reducing the extensibility in line 7, and promoting deformation at break in line 11. Line 11 also showed the highest cellulose-to-lignin ratio, which may explain its superior tensile strength. In addition, this line exhibited the highest accumulation of PHA, possibly further contributing to the stem mechanical performance. Thus the genetic modification influenced the cell wall chemical composition. Further research concerning spectroscopic characteristic of generated flax plants was performed.

### Spectroscopic analysis of transgenic flax plants

**Fig. 5:**
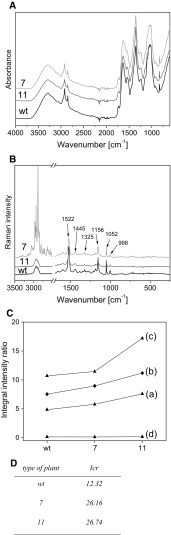
Spectroscopic analysis of analysed flax plants. **a** FTIR spectra of modified flax producing mcl-PHA (numbered as 7 and 11) and untransformed flax plants. **b** Raman spectra of the wt, 7 and 11 samples. **c** The integral intensity ratios of the IR bands at 1151/2920 cm^−1^ (a), 1047/2920 cm^−1^ (b), 988/2920 cm^−1^ (c) and 890/2920 cm^−1^ (d) for the control (wt), 7 and 11 samples. D. Crystallinity index (Icr) calculated for transgenic (#7, #11) and wild-type (wt) flax plants as the ratio between integral intensities of bands at 1380 cm^−1^ and 2920 cm^−1^

Spectroscopic methods, such as infrared (IR) and Raman spectroscopy were applied to investigate the structural properties of examined plants. The differences in cell wall composition were detected using these techniques. The contours in vibrational spectra typical for cellulose, lignin and pectin were observed (Fig. 5a, b) (Socrates 2001). Comparison of the integral intensities of the bands characteristic for cellulose with integral intensity of the band at 2920 cm^−1^ gives the following relationships for these ratios: wt < 7 < 11 samples (Fig. 5c). Such a result suggests that the wild-type sample exhibits the lowest content of cellulose in relation to the samples 7 and 11, which corresponds to biochemical data indicated that control plants had the lowest cellulose level, while transgenic plants were characterized by higher cellulose amount (Fig. 4).

Aromatic skeleton vibrations of lignin appear at about 1550 and 1510 cm^−1^. Lignin samples exhibit several weaker bands in the range below 1400 cm^−1^, originating from ν(C–C) + ν(C–O) + δ(C=O) coupled vibrations (1200–1300 cm^−1^), ν(ϕ) (ϕ-ring) vibration (1335 cm^−1^) aromatic δ(CH) + δ(C–O) (1000–1040 cm^−1^) as well as δ(CH) (900–920 cm^−1^) (Boeriu et al. 2004; Singthong et al. 2004; Sene et al. 1994). The most diagnostic spectral range that characterizes the lignin constituents is 1480–1560 cm^−1^ range of IR spectra. The bands in this range of IR spectra for control and transgenic samples could be deconvoluted into two Lorentzian components. The ratios of the integral intensities analyzed for these bands and the integral intensity of the 2920 cm^−1^ band for the wild-type and transgenic samples show that sample from plant numbered as 7 had the highest lignin amount (Fig. EMS 1A, B). The IR band at 1239 cm^−1^ may also be used for identification of the changes of lignin content in the samples (Dai and Fan 2011). Differences in the integral intensity ratio of the Raman bands at 1525/1445 cm^−1^ also show that concentration of lignin is the highest for plants of transgenic line #7, which confirmed biochemical results.

Another parameter, crystallinity index (Icr) is an important factor that characterizes the structural ordering of cellulose macromolecules (Bikales and Segal 1971). Obtained data showed that the Icr parameter calculated for the transgenic flax plants was over twofold higher than for control flax plants and its value was almost on the same level in plants obtained after transformation (Fig. 5d). It should be pointed out that these plants were characterized by the best mechanical properties and the values of the majority of measured mechanical parameters for modified plants were also more than twice as high as for control plants, wt.

Mechanical properties of plants are dependent on arrangement of cellulose microfibrils in cell walls and amounts of amorphous and crystalline regions in cellulose polymer (Cybulska et al. 2011). The values of mechanical parameters as Young’s modulus, tensile strength, hardness are proportional to the level of cellulose crystallinity (Lionetto et al. 2012). In fact this tendency is confirmed by present study, the calculated crystallinity index of cellulose for transgenic plants is highly correlated to mechanical properties. Thus the correlation coefficient between values obtained for Young’s modulus and Icr achieved high value of 0.99; it was also observed that correlation coefficient between tensile stiffness and Icr was equal to 0.92, and for tensile strength and Icr gave value 0.77.

Additionally the detection of mcl-PHA in transgenic and control flax lines was determined by FTIR method. The differences in the integral intensity ratios of the bands 1730/2920 and 1655/2920 cm^−1^ result from differences in the level of carboxyl groups of the hydroxy acids and pectins (Guo et al. 2013) (Fig. EMS 1C). The integral intensity ratio of the bands 1605/2920 cm^−1^ determines the level of pectins in the samples (Fig. EMS 1D). The data may indicate that amount of hydroxy acids in transgenic line 11 is the highest, thus spectroscopic analyses confirm biochemical data, obtained by GC-FID method (Fig. 1D).

### Effect of PHA synthesis on other genes

It is known that plants can modulate their transcriptional system, when they are in conditions of limitation or increase of different gene products (Zhai et al. 2016). Thus we analyzed the expression of selected genes in created flax plants (Fig. EMS 2). The results showed that expression of selected genes from hemicellulose, lignin, and polyamine metabolism pathways were changed in transgenic flax lines (7, 11) when compared to wild type flax cv. Nike (wt).

Since the modified plants showed changes in lignin content, the expression of a few genes from the monolignol biosynthesis pathway were tested. First the expression of the *PAL* (phenylalanine ammonia lyase) gene, which encodes the enzyme necessary for the first step of this pathway, was examined. The data showed a decrease in expression of this gene in line 11 and a raised level for line 7 when compared to wild-type plants. It should be pointed out that the enzyme encoded by this gene conducts the reaction of phenylalanine transformation to t-cinnamic acid, and it is the major enzyme from the phenylpropanoid pathway, which is the source of important second metabolites in plants, i.e. lignin, flavonoids, lignans, etc. Although observed overexpression of the *PAL* gene in plants from line 7 was not statistically significant it reflects higher lignin content in this line, and also the reduction in the expression of this gene in plants from line 11 confers a decrease of lignin level. The increase in expression of another gene important for lignin synthesis, i.e. *SAD* (sinapyl alcohol dehydrogenase), in line 7 and a reduction in line 11 in comparison to wild-type flax plants were also noted. Elevated expression of *SAD* gene in line 7 was statistically important. Another investigated gene from the phenylpropanoid pathway, i.e. CCoAOMT (caffeoyl-CoA O-methyltransferase), exhibited increased expression in plants from both tested lines. In line 7 expression of this gene was about 20%, while in plants from line 11 it was about 5%, but these changes were not statistically significant. CCoAOMT catalyzes methylation of caffeoyl-CoA to feruloyl-CoA (Zhong et al. 2000) and 5-OH-feruloyl-CoA to sinapoyl-CoA (Shi et al. 2010), giving the precursors for lignin synthesis. Although the changes in lignin biosynthesis genes *(SAD, CcoAOMT*) were not important from statistically point of view (except *SAD* in line 7), they are in accordance with chemical and spectroscopic analysis of lignin content in the tested flax plants.

What is very interesting, the expression of the gene from polyamine metabolism was also altered; thus higher (about 21%) expression of the SPDS (spermidine synthase) gene was noted in plants from line 7 in comparison to wild-type, untransformed plants. SPDS catalyzes the synthesis of spermidine from putrescine. It should be pointed out that polyamines play a role in plants in stresses by enhancing their tolerance to heavy metals, salt stress or osmotic stress (Gill and Tuteja 2010).

The expression of other genes connected with pectin metabolism (GAU1, alpha-1,4-galacturonosyltransferase 1) or hemicellulose metabolism (GMT, glucomannan 4-beta-mannosyltransferase 9-like; XXT, xyloglucan xylosyltransferase) was also measured, but only gene for XXT in line 7 exhibited significant increase when compared to wild-type plants. All these results show that transformation caused changes in the level of metabolites present in the plant cell wall.

## Conclusion

Plants are able to produce about 200 different fatty acids (Thelen and Ohlrogge 2002). Most studies are focused on changing the fatty acid metabolism with the aim of improving food application (Singh et al. 2005). Our investigations examined the possibility of polyhydroxy acid synthesis in flax to improve the plant mechanical properties. Production of mcl-PHA caused insignificant changes in fatty acid contents in modified flax plants and alterations in chemical composition of the plant cell wall measured by biochemical and spectroscopic methods. The expression of selected genes, encoding enzymes which are related to the cell wall metabolites, was also changed when compared to unmodified flax plants. The introduced modification improved significantly mechanical properties of whole flax stems, which corresponded to changes in biochemical composition of the cell wall. It was also shown that cellulose crystallinity was increased in generated flax plants over-expressing PHAC1 synthase when compared to wild-type, non-transformed plants and the value of this parameter was highly correlated with detected changes in Young’s modulus, tensile strength and tensile stiffness. The previous study concerning PHB synthesis in transgenic flax showed improvement of biochemical properties of whole stems, cultivated in field conditions and that observed increase was in the range 48–102%, these changes were however not noticed when fibres isolated from PHB producing flax plants were characterized (Wróbel-Kwiatkowska et al. 2009). In present research increase in mechanical properties of flax plants with over-expression of PHAC1 synthase was more than two-fold when compared to control flax plants, thus manipulation of this gene gave better results than expression of genes necessary for PHB synthesis. Further analysis is needed in the future to determine the impact of mcl-PHA synthesis on the mechanical characteristics of fibres and stems, derived from field-cultivated plants.

It would also be interesting to investigate the seeds of generated plants, especially in terms of the level and composition of fatty acids, which are accumulated as storage material in glyoxysomes.

## Electronic supplementary material

Below is the link to the electronic supplementary material.

A. The differences in the integral intensity ratio of the IR bands at 1545/2920 cm^−1^ (a), 1520/2920 cm^−1^ (b) and 1239/2920 cm^−1^ (c) for the control (wt), 7 and 11 samples. B. The differences in the integral intensity ratio of the Raman bands at 1525/1445 cm^−1^. C. The differences in the integral intensity ratio of the IR bands at 1730/2920 (a) and 1655/2920 cm^−1^ (b) for control (wt), 7 and 11 samples. D. The differences in the integral intensity ratio of the IR bands at 1605/2920 cm^−1^ for the control (wt), 7 and 11 samples (DOCX 78 kb)

Expression of selected genes examined in modified flax plants (#7 and #11) and control, wild-type plants (WT). Semi-quantitative PCR reaction was performed on a cDNA template as described in the Materials and methods section. Obtained products of PCR were detected on agarose gel (0.8%) and measured by densitometry. Quantitative data were normalized with expression of the actin gene, monitored in each transgenic line or control plants. The statistical analysis was performed by Student’s *t* test (*P < 0.05, **P < 0.01) (PDF 95 kb)
