# Supplementary figures and images for: Effect of mcl-PHA synthesis in flax on plant mechanical properties and cell wall composition
Source: Transgenic Res. 2018 Nov 27;28(1):77–90. doi: 10.1007/s11248-018-0105-y (PMC6353814; doi:10.1007/s11248-018-0105-y)

Relative expression of chosen genes

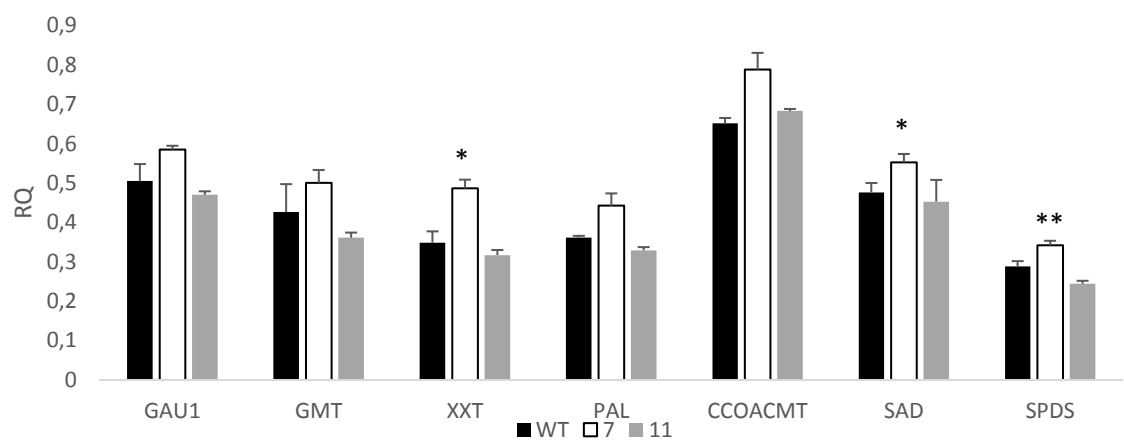

Supplement: Supplementary file 2 — Expression of selected genes examined in modified flax plants (#7 and #11) and control, wild-type plants (WT). Semi-quantitative PCR reaction was performed on a cDNA template as described in the Materials and methods section. Obtained products of PCR were detected on agarose gel (0.8%) and measured by densitometry. Quantitative data were normalized with expression of the actin gene, monitored in each transgenic line or control plants. The statistical analysis was performed by Student’s t test (*P < 0.05, **P < 0.01) (PDF 95 kb) [file 11248_2018_105_MOESM2_ESM.pdf]
